# Supplementary material for: Monoterpene Synthase Genes and Monoterpene Profiles in Pinus nigra subsp. laricio
Source: Plants (Basel). 2022 Feb 6;11(3):449. doi: 10.3390/plants11030449 (PMC8838282; doi:10.3390/plants11030449)
Supplement: Supplementary file 1 [file plants-11-00449-s001.zip › plants-1571914-supplementary.pdf]

Additional tables

Table S1. Full-length cDNA sequences of putative MBOSs and MTPSs in *Pinus* spp. retrieved from the NCBI database

Species	Function	Abbreviation	Accession mRNA sequence	ORF (bp)	Accession protein sequence	Amino acids (aa)
Pinus arizonica var. cooperi	2-methyl-3-buten-2-ol synthase	Par MBOS1	JN039226	1845	AFJ73545	614
Pinus banksiana	(-)-α pinene synthase	Pb MTPS1	JQ240304	1890	AFU73856	629
	(-)-β-pinene synthase	Pb MTPS2	JQ240291	1887	AFU73843	628
	(-)-β-pinene synthase (TPS-(-)Bpin2)	Pb MTPS3	JQ240292	1884	AFU73844	627
	(-)-α/β-pinene synthase	Pb MTPS4	JQ240290	1872	AFU73842	623
	α terpineol synthase	Pb MTPS5	JQ240308	1881	AFU73860	626
	(+)-3-carene synthase	Pb MTPS6	JQ240306	1881	AFU73858	626
	(+)-3-carene synthase	Pb MTPS7	JQ240305	1881	AFU73857	626
	(+)-α pinene synthase	Pb MTPS8	JQ240298	1887	AFU73850	628
	Monoterpene synthase	Pb MTPS9	JQ240296	1887	AFU73848	628
	Monoterpene synthase	Pb MTPS10	JQ240297	1887	AFU73849	628
	(-)-β-phellandrene synthase	Pb MTPS11	JQ240302	1866	AFU73854	621
Pinus contorta	(-)-α pinene synthase	Pc MTPS1	JQ240303	1890	AFU73855	629
	(-)-β-pinene synthase	Pc MTPS2	JQ240293	1884	AFU73845	627
	Monoterpene synthase	Pc MTPS3	JQ240294	1884	AFU73846	627
	(+)-3-carene synthase	Pc MTPS4	JQ240307	1881	AFU73859	626
	(+)-α pinene synthase	Pc MTPS5	JQ240295	1887	AFU73847	628
	α terpineol /1,8-cineole synthase	Pc MTPS6	JQ240309	1851	AFU73861	616
	(-)-camphene / (+)-α-pinene synthase	Pc MTPS7	JQ240299	1860	AFU73851	619
	(-)-β-phellandrene synthase	Pc MTPS8	JQ240301	1866	AFU73853	621
	(-)-β-phellandrene synthase	Pc MTPS9	JQ240300	1875	AFU73852	624
Pinus kesiya var. langbianensis	Monoterpene synthase	Pk MTPS1	KX394684	1956	AQZ36562	651
	α-pinene synthase	Pk MTPS2	KM382173	1875	AIY22674	624
Pinus massoniana	(-)-α pinene synthase	Pm MTPS1	KF547035	1890	AGW25369	629
	α-terpineol synthase	Pm MTPS2	KJ803197	1863	AIL88641	620
Pinus pinaster	α-pinene synthase	Pp MTPS1	KP780394	1890	ALB78130	629
	α-pinene synthase	Pp MTPS2	KP780395	1890	ALB78131	629
Pinus pinea	α-pinene synthase	Ppinea MTPS1	KR011842	1890	ALD18902	629
	α-pinene synthase	Ppinea MTPS2	KR011841	1890	ALD18901	629
Pinus pseudostrobus	2-methyl-3-buten-2-ol synthase	Pps MBOS1	JN039254	1845	AFJ73572	614
Pinus pseudostrobus var. estevezii	2-methyl-3-buten-2-ol synthase	Pest MBOS1	JN039251	1845	AFJ73569	614
Pinus sabiniana	2-methyl-3-buten-2-ol synthase	Psab MBOS1	JF719039	1845	AEB53064	614
Pinus tabuliformis	α-pinene synthase	Ptab MTPS1	EF608499	1890	ABY65904	629
Pinus taeda	(-)-α-pinene synthase	Pt MTPS1	AF543527	1890	AAO61225	629
	α-terpineol synthase	Pt MTPS2	AF543529	1884	AAO61227	627
	(+)-α-pinene synthase	Pt MTPS3	AF543530	1887	AAO61228	628
Pinus teocote	2-methyl-3-buten-2-ol synthase	Pteo MBOS1	JN039258	1845	AFJ73576	614
Physcomitrella patens	*ent*-kaurene synthase	Pt TPS-entKS	AB302933	2646	BAF61135	881

Table S2. Forward and Reverse primers used for the isolation of cDNAs and genomic sequences coding for MBOS (Group 1) and MTPSs (Groups 2-7) in *Pinus nigra* subsp. *laricio*. RACE, Rapid Amplification of cDNA Ends

	**Phylogentic group**	**Forward primers 5'→3'**	**Reverse primers 5'→3'**
**cDNA sequences**	Group 1	F1c: TCATCATTCCAACCTCTGGGA (165)	R1c: AGGCACAGGCTCAATGAC (1839)
	Group 2	F1c: CACCATGTGTTTGACAGCCC (147)	R1c: TTTATTTCATTGGTGGCGACG (1838)
	Group 3	F1c: TTCTAACCTGTGGGACGACAA (207)	R1c: TACATTAGCACGGGTTCG (1880)
	Group 4	F1c: TTCTGTCAACGCCTTATGGG (242)	R1c: TATAAAGGCACAGGTTCAAGGAG (1883)
	Group 5	F1c: GGACCGCCGTATCTGATGATG (167)	R1c: GTGACAGGATCAATGACGGT (1883)
	Group 6	F1c: CGCAACGTCTTATGAGGCAC (240)	R1c: CACAGGTTCAAGGACGGT (1851)
	Group 7	F1c: GGGAGGGAAATCCATAGCAC (120)	R1c: GCACAGTTTCAACGACGG (1879)
		**Race 5'**	**Race 3'**
**Race 5'-3' ends**	Group 1	R1: AACACATCTGAAGACACCGGG (545)	F1Race3': AATAGACTTTCCTTCGAGGC (1306)
		R2: CCATAAGGCGTTGAGAGGGAC (221)	
		R3: GGTTATCCGACACATGCGG (126)	
	Group 2	R1: AAGGCCCAGTGCAGTTGAGT (534)	F1Race3': CAGATAGTGCCCGTGGAGAA (1212)
		R2: GGCGCCTCATAATGTGTGG (236)	
		R3: GCATCTGACACAGCGGTTGTT (149)	
	Group 3	R1: CGCCAGAGCAGTTGTGTTGAG (534)	F1Race3': GCATTGACATGGTGCCTGAAC (1055)
		R2: CAGCATGCTCACCGTACGA (283)	
		R3: GTGGCCGAACTCATCCTGAT (161)	
	Group 4	R1: GAAGAGTTCGAAAGCCCAAGG (553)	F1Race3': GCAATTAAGAGATGGGATCCG (1189)
		R2: GCACGTTCACGGTAAGCGA (296)	
		R3: ACTGATTTCCGTGGCCTCG (140)	
	Group 5	R1: GGTTCGAAGACCCAAGGCAG (552)	F1Race3': AAGAGATGGGATCCGTCG (1198)
		R2: TCTCAGCACGTTCCCGATAAG (295)	
		R3: GCTCATGGAAGCACGTGTCA (156)	
	Group 6	R1: CAGCACTTTGTCTCCCACGTC (508)	F1Race3': CCGTCGGTTGTAGATTGTC (1186)
		R2: TCGGCACGTTTGAGGTAGG (287)	
		R3: ACTCACGCGAATGGAAGGTC (153)	
	Group 7	R1: TCTCTGCCACGTCCAATG (503)	F1Race3': CTTCGGAACAATGGAGGAGC (1152)
		R2: AGGCTATGGAATCATCGTCC (244)	
		R3: AGGTGCTATGGATTTCCCTC (141)	
		**Forward primers 5'→3'**	**Reverse primers 5'→3'**
**Genomic sequences**	Group 1	F1g: ATGTCTCTGCTCTCTGTCGC (1)	R1g: AGGCACAGGCTCAATGAC (1839)
	Group 2	F1g: ATGGCTCTACTTTCTGTCGC (1)	R1g: TTTATTTCATTGGTGGCGACG (1838)
	Group 3	F1g: ATGTCTCTTATTTCCGCTGTG (1)	R1g: TACATTAGCACGGGTTCG (1880)
	Group 4	F1g: ATGGATTTAATATCTGTC (1)	R1g: TATAAAGGCACAGGTTCAAGGAG (1883)
	Group 5	F1g: ATGTCTCCTGTTTCTGTGATC (1)	R1g: GTGACAGGATCAATGACGGT (1883)
	Group 6	F1g: ATGGCTCTGGCTCTGGTT (1)	R1g: CACAGGTTCAAGGACGGT (1851)
	Group 7	F1g: ATGGCTCTGGTTTCTGCTG (1)	R1g: GCACAGTTTCAACGACGG (1879)

Table S3. Statistical evaluation of the differences (one-way ANOVA followed by Tukey test) among the relative expression levels of *MBOS* and *MTPS* genes in five different tissues of *P. laricio*. The expression data of each gene were normalized using the geometric average of the two reference genes *CYP* and *upLOC*. Relative expression levels of the different *MBOS* and *MTPS* genes were referred to a calibrator, set to the value 1, which was represented by the gene in the five tissues with the lowest expression (*MTPS3* in YN). Different letters denote significant differences according to the Tukey's test ($p < 0.01$). YN: young needles; MN: mature needles; LS: bark and xylem combined from the leader stem; IS: bark and xylem combined from the interwhorl stems; R: roots.

MBOS 1 _LS	218,873 [a]	± 28.42
MTPS 6_LS	185,546 [a]	± 21.82
MTPS 5_LS	180,853 [a]	± 22.09
MBOS 1_MN	137,811 [b]	± 14.98
MBOS 1_R	129,661 [b]	± 13.33
MTPS 1_YN	120,568 [bc]	± 14.55
MTPS 7_LS	120,355 [bc]	± 14.52
MTPS 4_LS	113,445 [bc]	± 12.97
MTPS 4_R	105,215 [bc]	± 11.61
MTPS 6_IS	98,0454 [c]	± 10.89
MTPS 6_MN	95,839 [c]	± 9.06
MTPS 5_MN	82,6652 [c]	± 10.08
MTPS 2_LS	45,5297 [d]	± 5.83
MBOS 1_IS	40,0133 [d]	± 5.27
MTPS 7_MN	38,7512 [d]	± 4.74
MTPS 6_R	36,3261 [de]	± 4.23
MTPS 2_R	32,1126 [de]	± 4.86
MTPS 3_LS	27,2339 [de]	± 3.80
MTPS 5_YN	27,1918 [de]	± 3.43
MTPS 5_R	21,3925 [de]	± 3.00
MTPS 4_MN	17,3541 [ef]	± 2.80
MTPS 6_YN	15,1923 [ef]	± 2.90
MTPS 2_MN	14,0932 [f]	± 2.76
MTPS 4_IS	13,318 [f]	± 2.56
MTPS 5_IS	9,31126 [f]	± 2.20
MTPS 7_R	4,37264 [g]	± 1.18
MTPS 7_YN	4,23804 [g]	± 1.37
MTPS 7_IS	3,66427 [g]	± 1.26
MTPS 4_YN	3,40483 [g]	± 1.16
MTPS 2_IS	2,39381 [g]	± 0.89
MTPS 3_IS	2,31444 [g]	± 0.77
MTPS 3_R	2,25211 [g]	± 0.66
MTPS 2_YN	1,95116 [g]	± 0.43
MTPS 3_MN	1,2958 [g]	± 0.38
MTPS 3_YN	1 [g]	± 0.30

Table S4. List of primer pairs of *P. laricio MBOS*, *MTPS* and reference (*CYP* and *upLOC*) genes used in qRT-PCR analyses

Gene	Forward primer (5'-3')	Reverse primer (5'-3')
MBOS1	GCAATGTTCCAATCACTTCCAAG	AGGCACAGGCTCAATGACG
MTPS2	GCTTCTCAAACCAGACTGC	TTGGTGGCGACGCTGTAG
MTPS3	TCTCGGGTCAACACAGGAAG	CCATCTCGGTAGTTGTAGAAGTG
MTPS4	CTGGAGCAACAGAGGAAGATG	GTATTTGTAACCGTAATGGAAAGC
MTPS5	GCCCGTGGAGAAGAAGC	TGGGAACGCTGCTGTTTG
MTPS6	TTAAACCCGACAGCAATGTTCCC	GTGGCATCACCGTAACCATCTC
MTPS7	TGCTGTCAATCAAGTCAATGC	GCAACACTGAAGCCATCTCTG
CYP	TGTAGAGGGCTTGGAGGTC	CAAGCGAGCTGTCCAGAGT
upLOC	GGTTTGCTTTGGAGGATATG	GTCCAATGTGCACCTCGT

Additional figures

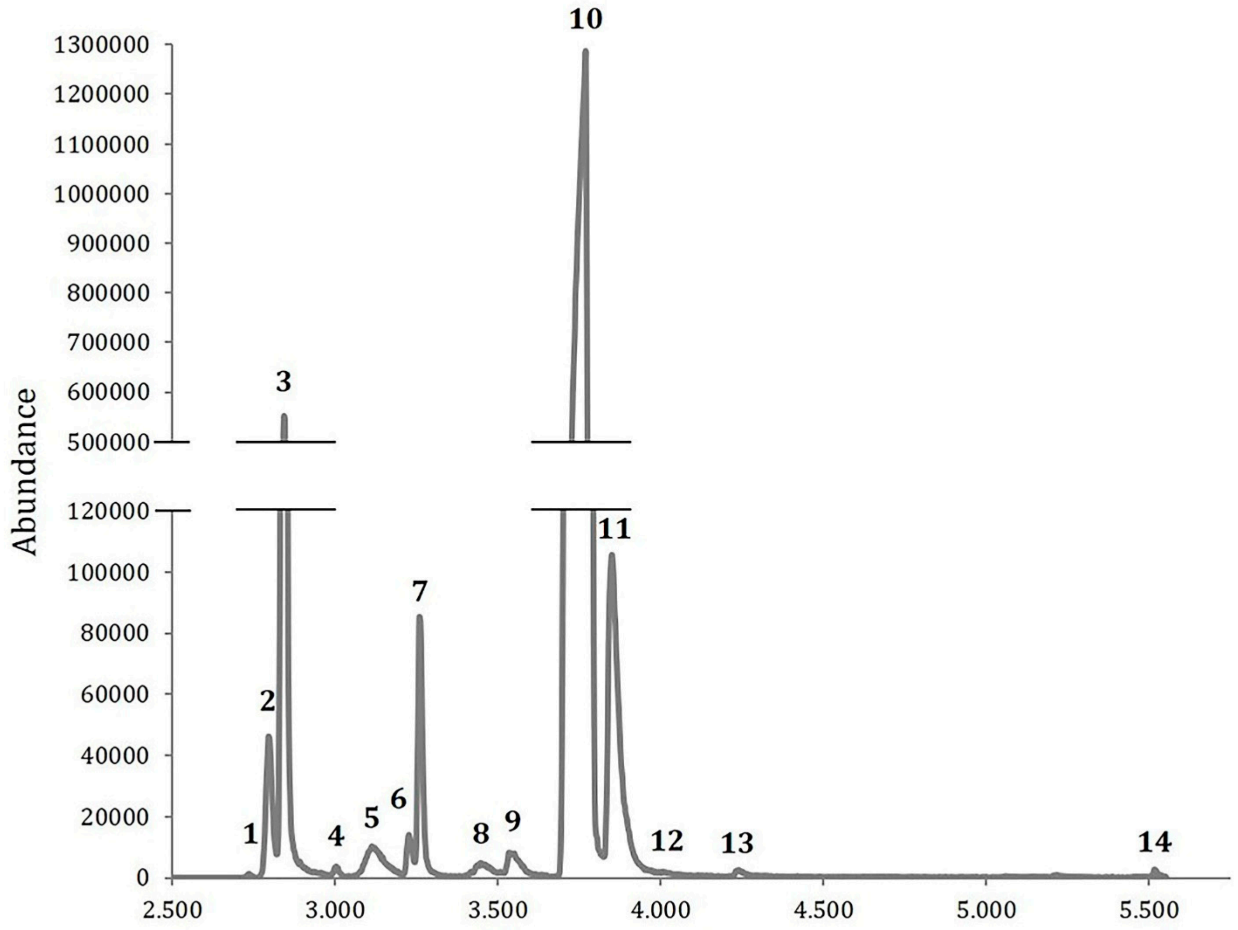

Figure S1. A representative GC-MS profile of the monoterpenes extracted from the young needles of *P. laricio*. Single ion monitoring at m/z 136, 121 and 93. (1) tricyclene, (2) α-thujene, (3) α-pinene, (4) α-fenchene, (5) camphene, (6) sabinene, (7) β-pinene, (8) myrcene, (9) δ-3-carene, (10) β-phellandrene, (11) limonene, (12) terpinolene, (13) α-terpineol and (14) bornyl acetate.

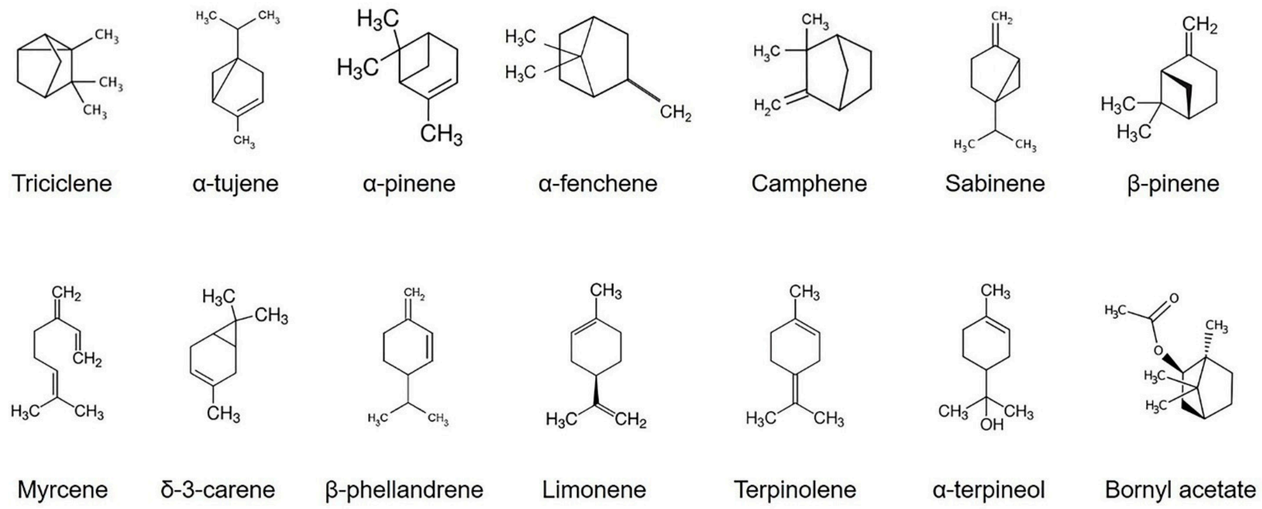

Figure S2. Chemical structures of the monoterpenes found in different tissues of *P. laricio*.

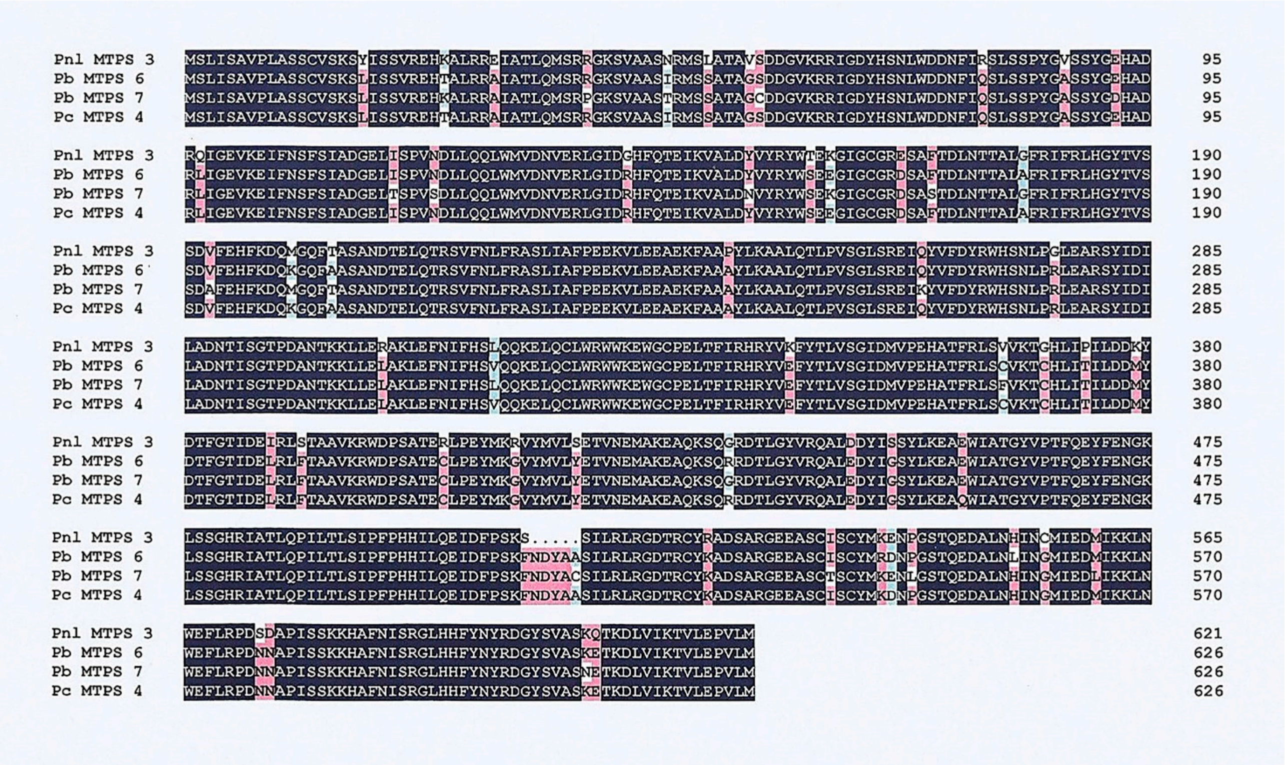

Figure S3. Alignment of deduced amino acid sequences of MTPSs belonging to the phylogenetic group 3. Amino acid residues with blue background indicate highly conserved regions, while amino acid residues which are identical in more than 50% of the proteins are in pink background. Pb, *Pinus banksiana*; Pc, *Pinus contorta*; Pnl, *Pinus nigra* subsp. *laricio* (*P. laricio*).

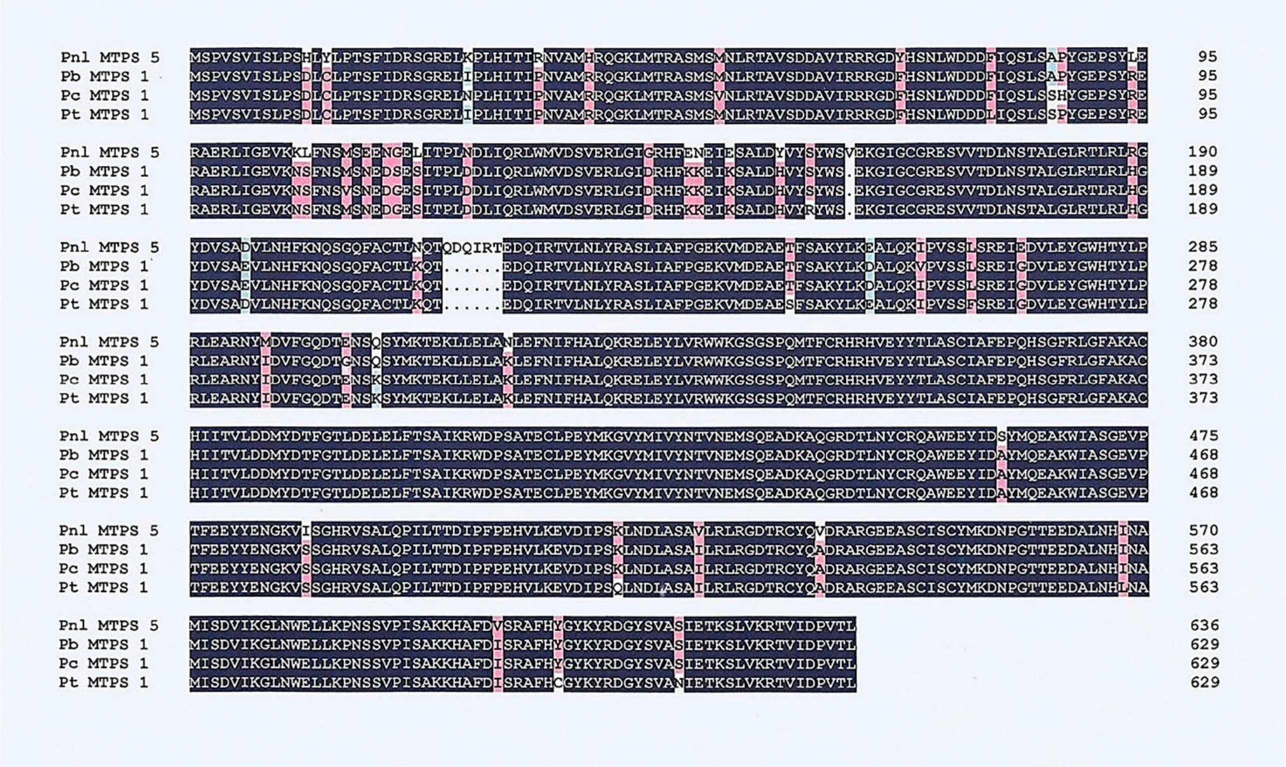

Figure S4. Alignment of deduced amino acid sequences of MTPSs belonging to the phylogenetic group 5. Amino acid residues with blue background indicate highly conserved regions, while amino acid residues which are identical in more than 50% of the proteins are in pink background. Pb, *Pinus banksiana*; Pc, *Pinus contorta*; Pt, *Pinus taeda*; Pnl, *Pinus nigra* subsp. *laricio* (*P. laricio*).
